# Detection of Three Opioids (Morphine, Codeine and Methadone) and Their Metabolites (6-Monoacetylmorphine and 2-Ethylidene-1,5-dimethyl-3,3-diphenylpyrrolidine) in Larvae of *Lucilia sericata* Species by UHPLC-TF-MS and Validation

**DOI:** 10.3390/molecules28124649

**Published:** 2023-06-08

**Authors:** Erika Buratti, Gianmario Mietti, Marta Cippitelli, Alice Cerioni, Rino Froldi, Mariano Cingolani, Roberto Scendoni

**Affiliations:** Forensic Medicine Laboratory, Institute of Legal Medicine, University of Macerata, 62100 Macerata, Italy; burattierika@gmail.com (E.B.); gianmario.mietti@gmail.com (G.M.); cippitelli.marta@gmail.com (M.C.); rino.froldi@unimc.it (R.F.); mariano.cingolani@unimc.it (M.C.); r.scendoni@unimc.it (R.S.)

**Keywords:** morphine, methadone, codeine, 6-MAM, EDDP, larvae, forensic entomotoxicology, UHPLC-FT-MS, post-mortem, *Lucilia sericata*

## Abstract

Insects on corpses could be a useful tool for the detection of exogenous substances such as drugs of abuse. The identification of exogenous substances in carrion insects is critical for proper estimation of the postmortem interval. It also provides information about the deceased person that may prove useful for forensic purposes. High-performance liquid chromatography coupled with Fourier transform mass spectrometry is a highly sensitive analytical technique that can identify substances even at very low concentrations, such as in the case of searching for exogenous substances in larvae. In this paper, a method is proposed for the identification of morphine, codeine, methadone, 6-monoacetylmorphine (6-MAM) and 2-ethylidene-1,5-dimethyl-3,3-diphenylpyrrolidine (EDDP) in the larvae of *Lucilia sericata*, a common carrion fly widely distributed in temperate areas of the world. The larvae, which were reared on a pig meat substrate, were killed once they reached their third stage by immersion in hot water at 80 °C and aliquoted into 400 mg samples. The samples were fortified with 5 ng of morphine, methadone and codeine. After solid-phase extraction, the samples were processed with a liquid chromatograph coupled to a Fourier transform mass spectrometer. This qualitative method has been validated and tested on larvae from a real case. The results lead to the correct identification of morphine, codeine, methadone and their metabolites. This method could prove useful in cases where toxicological analysis must be conducted on highly decomposed human remains, where biological matrices are very limited. Furthermore, it could help the forensic pathologist to better estimate the time of death, as the growth cycle of carrion insects can undergo changes if exogenous substances are taken.

## 1. Introduction

The search for drugs on cadavers is one of the practices behind forensic toxicology. Chemical toxicological investigations on cadaveric material can be difficult to conduct if the remains are in an advanced state of decomposition or have been subjected to a strong action of external and putrefactive events. In these situations, when bodies are found even days after death, conventional biological matrices may no longer be available [1]. An interesting alternative source may be insects growing on the remains [2].

The first use of insects in toxicological analyses dates back to 1980, when Beyer and colleagues described the possibility of obtaining information about antemortem drug intake by the deceased through maggots grown on corpses [3]. This practice became increasingly popular and took the name of forensic entomotoxicology [4]. It has been shown in several studies that insect larvae are able to accumulate substances present in the body on which they grow, in particular morphine [5]. Necrophagous insects lay their eggs on the cadaver in the first hours after death. The eggs hatch and the larvae begin to grow and feed on the flesh of the bodies passing through three larval stages. At the end of the third instar, larvae enter their pupal stage, from which the adult flies will emerge after metamorphosis [6].

Over the years, a number of studies have been conducted on the ability of necrophagous insects to accumulate exogenous substances present in the bodies of the corpses they have fed on [7,8,9,10]. This feature makes possible the identification of substances through the analysis of insect larvae by different techniques, from immunoassays to liquid and gas chromatography tandem mass spectrometry [11,12,13].

The Orbitrap technique involves an electrostatic ion trap in which ions, once injected, begin to oscillate in complex spiral trajectories under the influence of the electrostatic field and with a frequency directly proportional to the *m*/*z* ratio. The mass spectrum obtained from the current induced by the oscillations of the ions and converted into frequency by Fourier transform is the ion intensity in relation to the value *m*/*z* [14]. The choice of the analytical instrument was induced by its higher analytical sensitivity with respect to other techniques and by the possibility to identify molecules on the basis of their molecular weight. This made it possible to operate using small amounts of sample and reagents.

**Figure 1:**
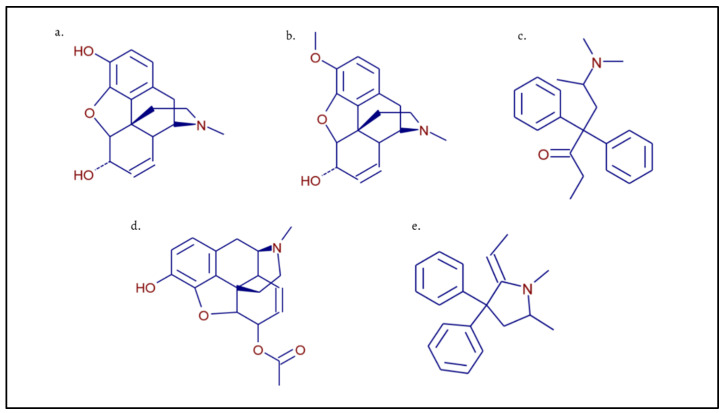
Chemical structures of target analytes and their metabolites: (**a**) morphine; (**b**) codeine; (**c**) methadone; (**d**) 6-monoacetylmorphine; (**e**) 2-ethylidene-1,5-dimethyl-3,3-diphenylpyrrolidine.

In this paper, a validation of a qualitative method is proposed for the simultaneous identification of three opioids and their metabolites: morphine, codeine, methadone, 6-monoacetylmorphine and 2-ethylidene-1,5-dimethyl-3,3-diphenylpyrrolidine (Figure 1), in the larvae of *Lucilia sericata*, by high-performance liquid chromatography (UHPLC) coupled to the high-resolution mass spectrometer Orbitrap (FT-MS).

Opioids were chosen as a target for this study because many drug-related deaths are due to morphine abuse [15] by drug addicts. Furthermore, in Italy, methadone is often administered as a replacement therapy. The larvae of *L. sericata* were reared on pig meat, then extracted and analyzed in UHPLC-FT-MS. The following method was applied to larvae taken from a real subject found days after death.

## 2. Results and Discussion

### 2.1. Validation

The qualitative validation studies followed the Standard Practices for Method Validation in Forensic Toxicology of the Scientific Working Group for Forensic Toxicology [16].

The recommended validation parameters included: interference studies, limit of detection (LOD), carryover assay and, since the analytical method was UHPLC-FT-MS, ionization suppression/enhancement evaluation. These tests were conducted to demonstrate the reliability of the identification method, and all provided satisfactory results.

#### 2.1.1. Limit of Detection

The limit of detection incorporates instrumental performance as well as the sample matrix and inherent procedural limitations. The LOD was assessed at 0.01 ng mg^−1^.

#### 2.1.2. Interference Studies

Regarding the evaluation of matrix-related interference, no interference signal was detected in the white samples. The recoveries for morphine, codeine and methadone were, respectively: 105%, 78% and 83%.

Since two deuterated internal standards (morphine-d3 and methadone-d3) were used for this method, interference studies related to the stable isotopes internal standards were also performed. No interference was detected between the internal standards and the target analytes.

Finally, with regard to the assessment of interference from other opioids and their metabolites commonly used in our laboratory (heroin, dihydrocodeine, hydromorphone, hydrocodone, oxymorphone, oxycodone, tramadol and fentanyl), the analyzed compounds showed different retention times than morphine, codeine, 6-MAM, methadone and EDDP.

#### 2.1.3. Ionization Suppression/Enhancement

Samples of neat standards and blank matrix samples described in Section 3 were injected to determine the average peak areas of the analytes. The measurement results reported values within the acceptable range of ±25%. These results confirm the usefulness of the SPE procedure for cleaning the sample before chromatographic injection, in order to obtain a reproducible and reliable result without major matrix interference.

#### 2.1.4. Carryover

Finally, no carryover was observed. This means that the applied method allows for complete elimination of any residual analytes, making it efficient for the analysis of morphine, codeine and methadone from larval samples.

### 2.2. Real Case Application Results

**Figure 2:**
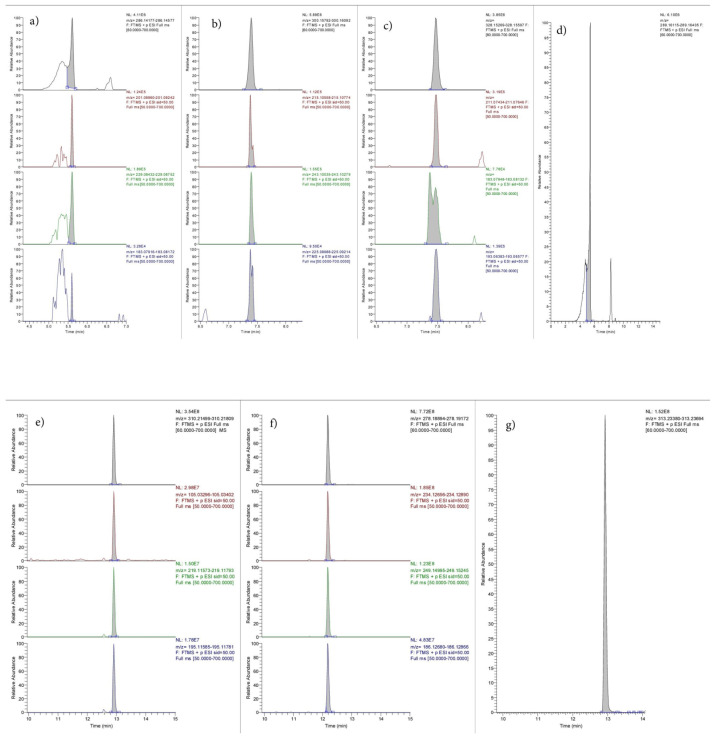
Chromatogram of morphine, codeine, 6-mam, morphine-d3 (IS), methadone, EDDP and methadone-d3 (IS), recognized by their EM and their PI (see Table 1), in larval samples (LS). Peak description: (**a**) morphine; (**b**) codeine; (**c**) 6-mam; (**d**) morphine-d3 (IS); (**e**) methadone; (**f**) EDDP; (**g**) methadone-d3.

**Figure 3:**
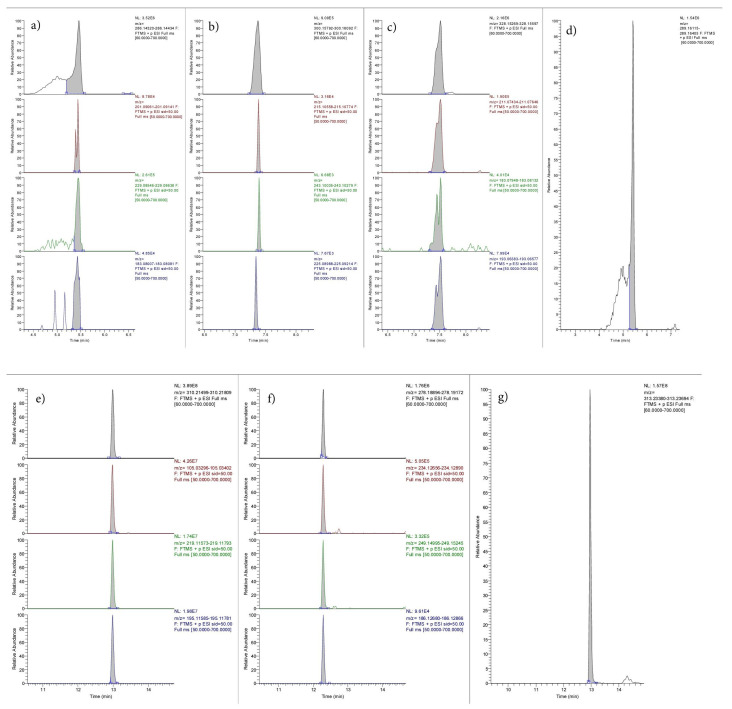
Chromatogram of morphine, codeine, 6-mam, morphine-d3 (IS), methadone, EDDP and methadone-d3 (IS), recognized by their EM and their PI (see Table 1), in hair samples (HS). Peak description: (**a**) morphine; (**b**) codeine; (**c**) 6-mam; (**d**) morphine-d3 (IS); (**e**) methadone; (**f**) EDDP; (**g**) methadone-d3.

To demonstrate the applicability of the method, larvae of *L. Sericata* were analyzed. The larvae were growing on the ocular cavities of a male corpse found in the summer season, four days after death, in an advanced state of decomposition. The remains were severely degraded, also because of the high temperatures that accelerated the post-mortem decay process; hence, bodily fluids were not available. Since the deceased had a known history of opiate addiction and was on methadone therapy, larvae from the eye cavities were collected to perform a toxicological analysis. The analysis was conducted both on real case larval samples (LS) and hair samples (HS) for a comparison of the results. Morphine, codeine and methadone were identified in both types of samples. Furthermore, the metabolites 6-monoacetylmorphine (6-MAM) and 2-ethylidene-1,5-dimethyl-3,3-diphenylpyrrolidine (EDDP) were detected, as shown in Figure 2 and Figure 3.

Figure 2 and Figure 3 show that the chromatographic analysis conducted on the two types of samples resulted in a good separation of the different analytes, with no overlapping chromatographic peaks in either the larvae or hair sample. The elution of the analytes occurred within sixteen minutes due to the applied gradient. Likewise, all peaks related to EMs showed satisfactory symmetry. The presence of morphine, codeine, methadone and their metabolites in the LS proves the presence of the substances in the tissues of the subject from which they were collected. Moreover, the presence of the same substances in HS confirms the results obtained and allows us to hypothesize habitual use of the substances.

### 2.3. Discussion

The fact that morphine, methadone and their metabolites were detected in the larvae indicates that the exogenous substances were actually taken by the deceased. The larvae were also collected from the ocular cavities, so it can be assumed that the immature flies also fed on vitreous humor. Since there is a direct correlation between the presence of morphine in the vitreous humor and blood [17], we can speculate that the deceased subject was under the narcotic effect of morphine and methadone at the time of death. From the perspective of quantitative analysis, substance concentrations have been calculated on several instances and for various substances including morphine, codeine, methadone and metabolites. The significant obstacle here is that there is a high variability in the concentration of drugs that have been quantified [18,19]. This fact could be due to the mechanism of detoxification and expulsion of exogenous substances by these organisms, mainly through the Malpighian tubules [20]. Thus, it is not possible to define a direct correlation between the concentration of the substance on the larvae and the amount of substance that may have been consumed by the subjects while still alive.

In interpreting the analytical result and thereby relating it to death, the context of all available and pertinent information must be well considered, including autopsy findings, drug tolerance, related cumulative toxic effects to therapy, variability in response, and pre-existing medical conditions.

Referring to this research, several authors have demonstrated the important role of an individual’s pre-existing disease state in determining susceptibility to overdose. Pre-existing pathological processes and tolerance make the concept of overdose not absolute [21]. Therefore, the evaluation of a death from an inappropriate intake of drugs, in particular opiates, must be evaluated based on the singular case. Often, having obtained a certain concentration of substance in conventional matrices (blood, urine) is not in itself a result which can be generalized, but it must be contextualized. However, the analysis on unconventional matrices is very important in cases where the remains are in an advanced state of decomposition; thus, conventional matrices are no more available. Different studies are already present in the literature for the detection of opioids in alternative matrices [22,23]. In unconventional matrices such as larvae, quantitative data could be even more difficult in relation to the mechanism of death. Furthermore, an additional variable to consider is the multiple consumption of different types of toxic substances, which may have cumulative and synergistic pharmacodynamic effects on larvae [24].

The use of entomotoxicology in connection with court cases has led to the development of a new branch of forensic entomotoxicology in recent decades because of broad scientific interest in this discipline. The main purposes of this discipline include, firstly, the possibility of detecting a variety of xenobiotics; secondly, the study of the influence that drugs have on the growth and development of the studied insect; and finally, the experimentation of different methods and approaches for the detection of xenobiotics [25].

In response to the interest from the scientific community that this field has developed, an increasing number of studies have been conducted over the past two decades [24]. In fact, through toxicological analysis carried out on necrophagous insects grown on the remains of a corpse, it is possible to obtain important information regarding drugs of abuse or drug therapies followed by the person whilst still alive. It has been shown that during their life cycle, larvae are able to accumulate within themselves xenobiotics present in the tissues of the remains on which they feed. Substances such as cocaine, morphine, codeine, methadone, phenobarbital, amitriptyline and nortriptyline have been detected in different developmental states of these organisms [26]. The ability to determine whether a living person had been using substances or was being treated with specific drugs can be very important in forensic toxicology, since it can help establish a more detailed circumstantial picture of the case.

Necrophagous insects not only accumulate substances within themselves as they feed, but sometimes their developmental processes are affected by the type of substance ingested. It has been shown in several studies that the normal developmental timing of growth stages undergoes alterations depending on the substances present in the substrate on which they feed. Lopes de Carvalho (2012), for example, showed that in the presence of cocaine, the third-stage larvae and the development of the puparial process of two species of Calliphoridae are greater and faster than under normal conditions [27]. On the other hand, Lui X. (2009) and Goff. M (1991) stated that in the presence of malathion and heroin, larval development is accelerated compared to normal conditions, while the puparial process is slowed down in *Chrysomia megacephala* and Diptera sarcophagidae [28,29]. For this reason, recognizing whether the larvae feed on remains containing exogenous substances would be of considerable importance to forensic pathologists to determine the post-mortem interval (PMI) with good approximation.

*Lucilia sericata*, also called sheep fly, is a widespread species of carrion fly—probably one of the most widespread Calliphoridae in the world—and is among the first colonists to lay eggs on a body after death. *L. sericata* is a species that has been studied by several research groups for its implications in forensic entomology and the estimation of PMI [30,31]. Although studies have shown that on different types of animal flesh the development of *L. sericata* is unchanged, Zhao et al., 2005, 2008, showed again that in the presence of morphine, the duration of larval development is shortened [32,33]. This confirms the importance of having a rapid and clear method for the determination of exogenous substances in necrophagous insects for correct determination of the time of death.

In today’s state of the art, as far as we can infer from the current literature, we can therefore say that the quantitative analysis of exogenous substances in carrion insects, including morphine, codeine and methadone, loses its importance for forensic purposes. However, identification of the type of substance found in the body of the larva is crucial information for determining the time of death. Based on this evidence, this is the reason why the authors proposed a validation of a qualitative method based solely on the identification of three opioids and their metabolites in the worldwide diffused species of diptera larva *Lucilia sericata*.

## 3. Materials and Methods

### 3.1. Reagents

Morphine, methadone, codeine, 6-MAM, EDDP standards, and morphine-d3 and methadone-d3 internal standards (IS) were purchased from Sigma Aldrich (Milan, Italy). For the extraction procedure, distilled water (H_2_O) for analysis, sodium hydroxide (NaOH), methanol (MeOH) for analysis (≥99.9%), hydrochloric acid for analysis (37%), dichloromethane for analysis stabilized with amylin (≥99.9%), 2-propanol (99.8%) and ammonium hydroxide (32%) of reagent grade were purchased from Carlo Erba (Cornaredo, Italy). For the UHPLC analysis, methanol for UHPLC-MS (99.99%), ultrapure water for UHPLC-MS and formic acid (FA) for LC-MS (≥99%) were purchased from Carlo Erba (Cornaredo, Italy). All reagents and laboratory consumables were stored according to the manufacturer’s instructions.

### 3.2. Instrumentation

For solid-phase extraction (SPE), Isolute HCX cartridges (10 mL capacity, 300 mg) were purchased from Biotage (Uppsala, Sweden). For qualitative analysis, the Thermo Scientific Dionex Ultimate 3000 chromatographic system (UHPLC) coupled with Thermo Exactive Plus Orbitrap (FT-MS) was used. For the chromatographic analysis, the column used was Kinetex Biphenyl 2.6 µm (50 × 2.1 mm) from Phenomenex with a flow set at 0.4 mL/min. The column temperature was set at 25 °C. The phases used were as follows: aqueous phase A: H_2_O + 0.1% FA; organic phase B: MeOH + 0.1% FA. Positive electrospray ionization (ESI) in selected ion monitoring (SIM) mode was performed for ions fragmentation. The gradient for the chromatographic analysis was the following: organic phase B from 2% to 100% in 9.5 min; maintenance of the gradient for 2 min; reversion to 2% B phase in 1 min and maintained for 2 min at 2% equilibrium.

### 3.3. Experimental Method

#### 3.3.1. Raising of *L. sericata* Larvae

In order to perform all the validation steps, *L. sericata* flies were captured in the field and, after being morphologically and genetically recognized, reared in a closed container with a net at a constant temperature of about 22 °C. The flies laid eggs on a minced pig meat substrate and were allowed to grow to their third stage. Once the third instar was reached, the larvae were collected and killed by immersion in heated water at 80 °C, divided into 400 mg aliquots and stored at −20 °C until analysis.

#### 3.3.2. Preparation of Samples

Aliquots of larvae (400 mg) spiked with morphine (5 ng), methadone (5 ng) and codeine (5 ng) standards were extracted by the addition of 2.5 mL of a solution of water and hydrochloric acid (*w*/*w* 9:1). IS solutions (20 ng), prepared from 1 ng µL^−1^ concentration solutions of morphine-d3 and methadone-d3, were added. The samples were incubated at 100 °C for 2 h, then allowed to cool down. The solutions were fortified with 3 mL of Tris buffer (pH 8) and the pH raised to 8.5 by the addition of NaOH. The samples were centrifuged and underwent the solid-phase extraction procedure.

#### 3.3.3. Solid-Phase Extraction and UHPLC Analysis

The solid-phase extraction procedure followed a method already employed in our laboratory for keratinous matrices, such as hair and nails, and adapted to larvae as well [1]. Briefly, after an initial activation with methanol and conditioning of the column with distilled water, the sample was run through it. This was followed by one wash with distilled water, one with a hydrochloric acid solution and a last one with methanol. The analytes were eluted with a dichloromethane/2-propanol solution (80:20) with 2% ammonium hydroxide.

The evaporated samples were resuspended with 50 µL of organic phase B, and 5 µL was injected into the UHPLC system. The analytical conditions are those described above. Ionized exact mass (EM) and produced ions (PI), obtained by collision-induced dissociation (50 eV), were used for the identification of morphine and methadone, all with an acceptance range of ±5 µg mL^−1^. The monitored values for the internal standards were, respectively: EM 289.16260 with retention time at 5.43 for morphine-d3; EM 313.23537 with retention time at 12.99 for methadone-d3. The analytes and their respective retention times are given in Table 1.

### 3.4. Validation Studies

#### 3.4.1. Limit of Detection

To assess the sensitivity of the method, the LOD was defined at 0.01 ng mg^−1^. To verify the reliability of the LOD, three different blank matrix samples were spiked with 0.01 ng of the three target substances and analyzed three times each.

#### 3.4.2. Interference

Interference studies were conducted on the matrix, on the IS, and on other analytes commonly used in the laboratory.

To evaluate the interference caused by the matrix effect, ten blank larvae samples were analyzed without the addition of IS.

To check the interference of the stable isotope internal standard, one aliquot of neat larvae sample was used as a blank matrix. Morphine-d3 (20 ng mg^−1^) and methadone-d3 (20 ng mg^−1^) were added to the blank sample and the signals of morphine and methadone were monitored. Similarly, another blank sample was fortified with 30 ng mg^−1^ of morphine and 30 ng mg^−1^ of methadone to monitor possible interferences with the IS due to the target analytes.

Finally, to evaluate the possibility of interferences caused by analytes commonly used in our laboratory, a solution containing common opioids and metabolites used in our laboratory (heroin, dihydrocodeine, hydromorphone, hydrocodone, oxymorphone, oxycodone, tramadol and fentanyl), at a concentration of 30 ng µL^−1^, was prepared.

#### 3.4.3. Ionization Suppression/Enhancement

To assess the suppression or enhancement of the ionization of targets, a post-extraction addition treatment was used. Low (0.5 ng µL^−1^) and high (30 ng µL^−1^) standard concentrations of pure standards were analyzed for methanol standards and for 60 mg of blank fortified nail samples. Neat standards were analyzed in six replicates. Samples of fortified matrix were analyzed in 10 replicates for each concentration. To be considered acceptable, values needed to be within ±25% suppression or elevation of ionization.

#### 3.4.4. Carryover

To assess the presence of carryover, a blank was analyzed immediately after analysis of a high concentration sample (30 ng µL^−1^). This measurement was performed three times.

### 3.5. Real Case Application

The larvae sample used for proof of applicability was collected from the oral and ocular cavities of a male cadaver. To assess the reliability of the method, the extraction was carried out both on LS and HS to confirm the results obtained from the larvae analysis.

## 4. Conclusions

In this study, we developed and validated a qualitative method for the identification of morphine, codeine and methadone in samples of *L. sericata* larvae. The method comprises solid-phase extraction followed by a sensitive UHPLC-FT-MS analysis. The utility of this method was confirmed through the analysis of larvae collected from a real case in which a body was found days after death. The efficiency of the extraction and the sensitivity of the analysis also allowed the identification of the main metabolites (6-MAM and EDDP) of the opioids sought. This method was able to aid the forensic toxicologist in understanding whether the remains belonged to a subject who used opioids. In addition, given the developmental adaptations of the larvae in relation to the exogenous substances they consumed, this method can assist forensic pathologists, adding useful tools to estimation of the time of death.

## Figures and Tables

**Table 1:** Retention times, ionized exact masses and produced ions used for identification of morphine, codeine, methadone, 6-MAM and EDDP.

	Morphine	Methadone	Codeine	6-MAM	EDDP
RT (min)	5.47	12.97	7.41	7.47	12.18
EM (*m*/*z*)	286.14377	310.21654	300.15942	328.15433	278.19033
IP (*m*/*z*)	201.09101	105.03349	215.10666	211.07540	234.12773
	229.08592	219.11683	243.10157	183.08040	249.15120
	183.08044	195.11683	199.07536	193.06480	186.12773

## Data Availability

The data underlying this article will be shared on reasonable request to the corresponding author.
